# Impact of electronic cigarette usage on the onset of respiratory symptoms and COPD among Chinese adults

**DOI:** 10.1038/s41598-024-56368-9

**Published:** 2024-03-07

**Authors:** Beibei Song, Honglin Li, Huiran Zhang, Libin Jiao, Siyu Wu

**Affiliations:** 1 The First Department of Pulmonary and Critical Care Medicine, The Second Hospital of Hebei Medical University/Hebei Key Laboratory of Respiratory Critical Care/Hebei Institute of Respiratory Diseases, No. 215 Heping West Road, Shijiazhuang, 050000 China; 2 Department of Biological Pharmacy, Hebei Medical University, Shijiazhuang, 050000 China; 3 Hebei Far East Communication System Engineering Company, Shijiazhuang, 050000 China; 4 The Third Department of Respiratory and Critical Care Medicine, the Second Hospital of Hebei Medical University, Shijiazhuang, 050000 China

**Keywords:** Electronic cigarette, Combustible cigarette, Respiratory symptoms, COPD, Chronic obstructive pulmonary disease, Risk factors

## Abstract

The prevalence of dual usage and the relatively low cessation rate among e-cigarette (EC) users suggest that ECs have not demonstrated significant effectiveness as a smoking cessation tool. Furthermore, there has been a substantial increase in the prevalence of EC usage in recent years. Therefore, the objective of this study is to investigate the association between EC use and the incidence of respiratory symptoms and chronic obstructive pulmonary disease (COPD). A total of 10,326 participants aged between 20 and 55 years, without any respiratory diseases or COPD, were recruited for the study. These individuals attended employee physical examinations conducted at 16 public hospitals in Hebei province, China from 2015 to 2020. Logistic regression models were utilized to assess the association between EC use and the risk of respiratory symptoms and COPD using risk ratios along with their corresponding 95% confidence intervals. Restricted cubic spline functions were employed to investigate the dose–response non-linear relationship. The robustness of the logistic regression models was evaluated through subgroup analyses, and sensitivity analyses. During the 5-year follow-up period, a total of 1071 incident cases of respiratory symptoms and 146 incident cases of COPD were identified in this cohort study. After adjusting for relevant confounding factors, EC users demonstrated a respective increase in the risk of reporting respiratory symptoms and COPD by 28% and 8%. Furthermore, dual users who used both ECs and combustible cigarettes exhibited an elevated risk of incident respiratory symptoms and COPD by 41% and 18%, respectively, compared to those who had never used non-users of any cigarette products. The association between daily EC consumption and the development of respiratory symptoms, as well as COPD, demonstrated a significant J-shaped pattern. The potential adverse association between the consumption of ECs, particularly when used in combination with combustible cigarettes, and the development of respiratory symptoms and COPD necessitates careful consideration. Policymakers should approach ECs cautiously as a prospective smoking cessation tool.

## Introduction

The available evidence suggests a significant surge in the popularity of ECs among adolescents and young adults worldwide in recent years^1^. Potential motivations for youth engagement in ECs encompass their enhanced sensory attributes, cost-effectiveness, and heightened accessibility and availability, thereby exposing them to the peril of developing nicotine addiction. The available evidence suggests that the utilization of EC was widely embraced in countries such as the United States and the United Kingdom^2,3^, while also experiencing significant growth in recent years in China. The findings from the China Chronic Disease and Nutrition Surveillance (CCDNS) surveys unveiled a notable surge in the weighted prevalence of current EC use among Chinese adults during 2015–2019. Among male participants, the latest prevalence of current EC users in 2019 was a remarkable 3.1% (95% CI 2.7–3.5), encompassing an astonishing 95% of the entire EC user population^4^. Furthermore, there is a substantial body of evidence indicating that young adults in China are emerging as a prominent and significant consumer demographic for EC^5–7^. The objective of our study was to examine the association between EC use and the incidence of respiratory symptoms and COPD among Chinese EC users.

The respiratory consequences of using combustible tobacco have been extensively recorded, while the impact on respiration from EC usage remains largely unexplored^8^. The clinical practitioners and researchers have increasingly focused on the respiratory symptoms linked to lung injury and COPD in connection with the utilization of EC^9,10^. The mortality rate of COPD in China surpasses 0.9 million on an annual basis, positioning it as the third most prevalent cause of fatality^11^. The findings of epidemiological studies conducted among young adults have indicated a significant association between the use of EC and the presence of respiratory symptoms^12,13^ as well as COPD^14,15^. However, long-term health effects of EC use are rarely available, as the evidence provided is limited to cross-sectional data or short-term studies.

Our study aimed to assess the association between EC use and respiratory symptoms as well as COPD, in individuals with or without concurrent combustible cigarette consumption, using a comprehensive longitudinal cohort dataset from 2015 to 2020.

## Methods

### Study sample

The data for this study were collected from employee physical examinations conducted in 16 designated public hospitals in Hebei province, China, during October 2015 and October 2020. The study involved 10,326 employees between the ages of 20 and 55 who had provided complete data on all essential factors. The participants hailed from diverse professional backgrounds, including office clerks, military veterans, firefighters, law enforcement officers, educators, medical practitioners and so on. Prior to participating in the survey for the first time, none of them had been diagnosed with any respiratory diseases. The 26 physical examinations, including blood and lung function tests, were completed by all participants in strict accordance with the Physical Examination Standards for Civil Servants of China^16^. The research was granted ethical approval by the Ethics Committee of the Second Hospital of Hebei Medical University. Written informed consent was obtained from all participants. The procedures adhered to the principles stated in the Declaration of Helsinki and relevant regulations.

### Definitions of health outcomes

According to the clinical characteristics of respiratory symptoms associated with EC use, respiratory symptoms were defined as the presence of chronic bronchitic symptoms for a duration of three months within the past year. These symptoms include daily and persistent coughing, any occurrence of wheezing, as well as congestion, phlegm production, or shortness of breath during light physical activity^17–19^. The absence of respiratory symptoms was determined based on the lack of any reported instances of the aforementioned symptoms.

The lung function test was a mandatory component of the employee physical examination process. All employees underwent the test, which was conducted by a respiratory physician following standardized protocols. Prior to the test, participants received guidance from physicians on practicing exhalations. Pre- and post-bronchodilator forced expiratory volume in one second (FEV_1_) and forced vital capacity (FVC) were measured as primary outcomes, with each employee's FEV1:FVC ratio calculated to assess the risk of incident COPD. The definition of new-onset COPD patients was based on the diagnostic criteria outlined by the Global Initiative for Chronic Obstructive Lung Disease (GOLD)^20^ and the expert consensus on COPD in China^21^, which were simultaneously considered in our analyses. The definition of new-onset COPD patients was established based on a post-bronchodilator FEV1: FVC < 70%, taking into account the patients' medical history as well as the findings from X-ray and biochemical examinations.

### Definition of status of cigarette smoking

Participants classified as non-users of EC and combustible cigarettes included those who indicated no prior experience with these products whatsoever. Participants who currently engage in the consumption of various forms of combustible tobacco products, such as traditional cigarettes, filtered cigarettes, hand-rolled cigarettes, homemade cigarettes, cigars, pipe tobacco, or water pipes on a daily basis or occasionally, will be categorized as current users of combustible cigarettes. Individuals who reported being ever smokers but had not quit smoking for more than a year were classified as current users; otherwise, they were categorized as never users. Participants categorized as EC users are those who reported regular use of ECs on a daily basis, intermittently, or one to two times per week. The cumulative duration of EC utilization was assessed through an additional questionnaire item asking participants, "*How many hours did you use electronic cigarettes per day*?" Individuals who reported ever use of ECs, but not exceeding once within a six-month period, were classified as current users; otherwise, they were categorized as never users. Dual users refer to individuals who reported simultaneous usage of both electronic and combustible cigarettes.

### Definitions of covariates

We obtained the covariates, encompassing demographic and physical measurement variables, from the employee physical examinations database. Demographic variables comprised participants' age, gender, education level (with completion of college or university considered as a high educational level), drinking habits, and marital status. Participants who consumed alcohol at least once a week were classified as drinkers. The physical examinations were conducted by physicians who underwent standardized measurement training and assessment provided by the 16 designated public hospitals in Hebei province^22^. According to the criteria set by the Working Group on Obesity in China (WGOC), participants are required to wear lightweight clothing and remove their shoes during weight and height measurements^23^. The body mass index (BMI) determined by dividing the weight in kilograms by the height in meters squared. The average of three readings obtained from electronic sphygmomanometers with identical specifications was used to determine blood pressure. Furthermore, we obtained blood samples from all participants following an overnight fast to assess their levels of fasting plasma glucose (FPG), total cholesterol (TC), triglycerides (TGs), and high-density lipoprotein cholesterol (HDL-C). The aforementioned covariates have been suggested to be associated with the development of respiratory symptoms and COPD in former studies^24–31^.

### Statistical analysis

The baseline characteristics were compared between individuals using ECs and those using combustible cigarettes. Categorical variables were presented as weighted percentages, accompanied by 95% CIs. Continuous variables were presented as weighted means, also with 95% CIs. The chi-square test was employed to compare differences among categorical variables at various levels, while the t-test was used for continuous variables. The response of “unable to answer” in the questionnaires was considered as missing data. The rate of missing data was below 5% and was addressed using multiple imputation techniques.

Logistic regression models were employed to estimate the association between EC use and the risk of respiratory symptoms and COPD. The odds ratio (OR) and 95% confidence intervals (CIs) were utilized as measures of EC use and the risk of incident respiratory symptoms and COPD. The ORs and 95% CIs were adjusted for relevant covariates, including demographic factors, as well as other confounding variables described above in the models. The RCS functions were utilized to examine the non-linear association between daily ECs consumption and the incidence risk of respiratory symptoms and COPD. The fit of nonlinear curves was optimized when 3 knots were incorporated into the models, this approach prevented accuracy reduction due to over-fitting^32^. In order to further validate the robustness of the correlation, we conducted the subgroup analysis by categorizing all potential covariates and the sensitive analysis by excluding part of participants. This comprehensive approach was employed to reevaluate the association between ECs usage and the incidence risk of respiratory symptoms as well as COPD. By incorporating various factors that could potentially impact the relationship, our aim was to provide a more precise and dependable estimation of this association.

All statistical analyses were performed in STATA software version 15.0 (StataCorp LLC, College Station, TX). Statistical significance was determined at a two-sided *p*-value < 0.05.

## Results

### Descriptive analyses

**Figure 1:**
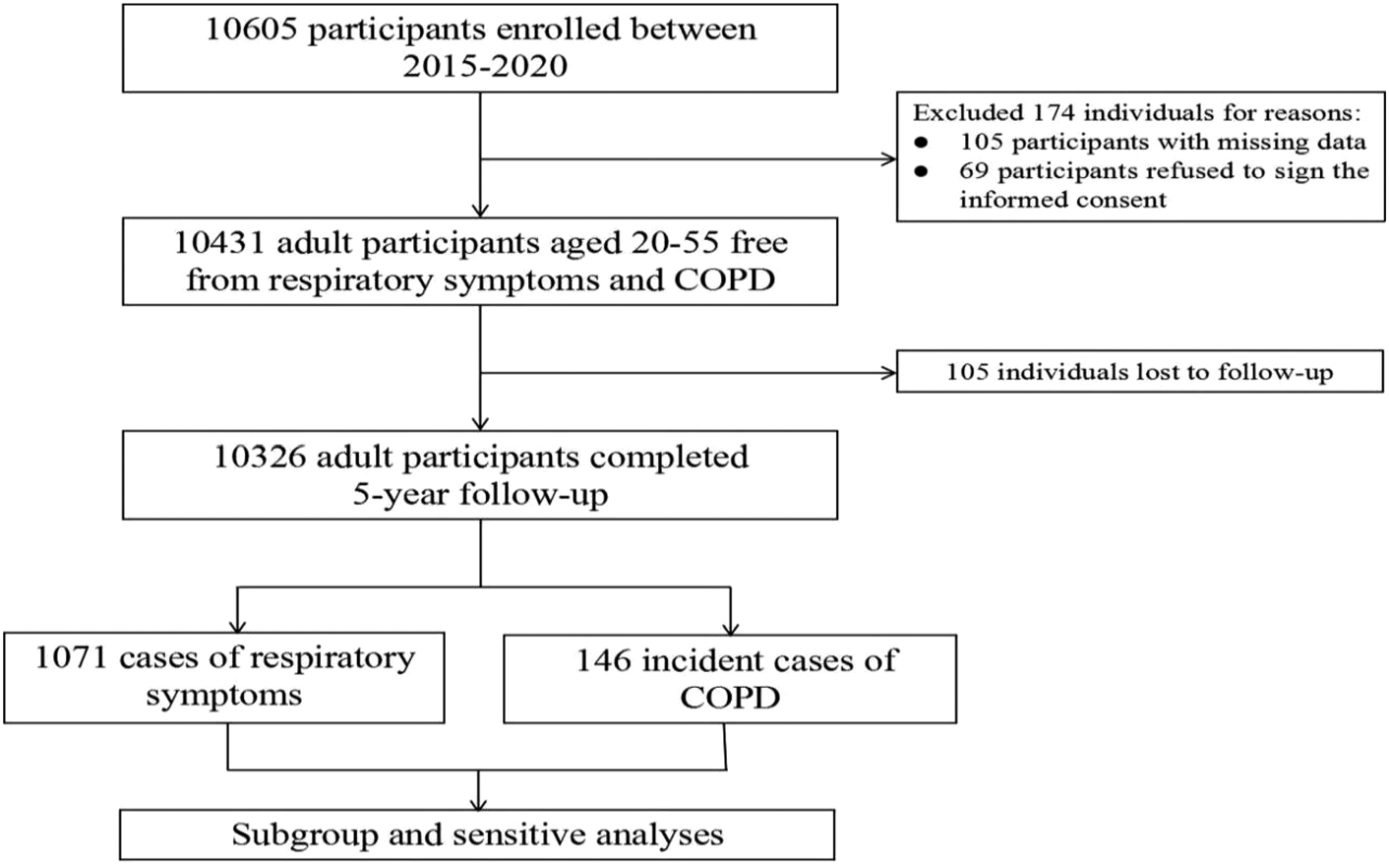
Flow chart illustrating the sample selection for the present study.

A total of 10,326 employees without any respiratory diseases underwent physical examinations in 16 designated public hospitals in Hebei province from October 2015 to October 2020. The enrollment process of participants in our study is illustrated in Fig. 1.

The study included 7104 non-users, 2879 combustible cigarette users, 156 EC users, and 187 dual users of EC and combustible cigarettes. The average age of current EC users was 43.3 (42.8–43.8) years old, with a median age of 38 years old. (Table 1).

**Table 1:** Baseline characteristics of study participants categorized based on their current cigarette usage status.

Characteristics	Current status of cigarette usage				*p*-value
	Non-users	Combustible cigarette users	EC users	Dual users	
Number of participants	7104	2879	156	187	
Age (years)	43.2(43.0–43.4)	42.6(42.0–43.3)	43.0(39.2–46.8)	44.1(42.0–46.2)	0.097
Male (%)	51.3(50.9–51.7)	96.4(95.9–96.9)	88.7(85.0–92.4)	89.9(88.0–91.8)	< 0.001
High education (%)	31.5(31.0–32.0)	32.4(31.1–33.7)	30.9(22.3–39.5)	31.8(29.6–34.0)	0.842
Drinking at present (%)	15.7(15.1–16.3)	16.8(15.9–17.7)	16.3(13.1–19.5)	16.5(14.1–18.9)	0.595
Currently married (%)	79.6(78.7–80.4)	76.3(75.2–77.4)	78.4(74.3–82.6)	77.1(74.8–79.4)	0.004
Physical measurements					
BMI (kg/m^2^)	23.3(22.5–24.2)	23.1(21.9–24.4)	22.9(20.3–25.7)	23.8(21.8–25.8)	0.207
SBP (mm Hg)	117.8(108.2–127.7)	119.9(109.3–130.9)	120.3(100.9–139.7)	118.9(101.8–136.0)	0.060
DBP (mm Hg)	71.0(65.4–76.6)	70.3(62.5–78.2)	72.2(55.6–88.9)	71.4(59.3–83.5)	0.084
FPG (mg/dl)	88.6(78.2–99.0)	89.2(70.3–108.1)	88.5(65.6–111.4)	88.3(73.2–103.4)	0.063
TG (mmol/L)	1.70(1.33–2.03)	1.67(1.26–2.09)	1.69(1.03–2.35)	1.61(1.15–2.07)	0.076
TC (mmol/L)	4.27(3.73–4.81)	4.21(3.30–5.12)	4.20(2.89–5.51)	4.23(3.14–5.32)	0.114
HDL-C (mmol/L)	1.10(0.91–1.30)	1.13(0.85–1.41)	1.11(0.62–1.60)	1.13(0.78–1.48)	0.121
FEV_1_ (L)	3.27(2.94–3.60)	3.18(2.77–3.59)	3.25(2.07–4.43)	3.15(2.28–4.02)	0.203
FVC (L)	3.10(2.84–3.37)	3.21(2.67–3.76)	3.20(2.21–4.19)	3.24(2.23–4.25)	0.093
FEV_1_/FVC (%)	93.6 (91.8–95.4)	94.1(92.0–96.2)	93.9(81.2–107.1)	94.1(83.3–104.9)	0.069

Non-users: individuals who never consumed any cigarette products; Dual users: individuals who concurrently engage in the consumption of both electronic cigarettes and combustible cigarettes.

EC, electric cigarette; BMI, body mass index; SBP, systolic blood pressure; DBP, diastolic blood pressure; FPG, fasting plasma glucose; TG, triglyceride; TC, total cholesterol; HDL-C, high-density lipoprotein cholesterol; FEV_1_, forced expiratory volume in one second; FVC, forced vital capacity; FEV_1_/FVC, the ratio of forced expiratory volume in one second and forced vital capacity.

### Association of EC and respiratory symptoms

The study identified a total of 1071 incident cases of respiratory symptoms, with non-users accounting for 191, combustible cigarette users accounting for 764, EC users accounting for 42, and dual users of combustible cigarettes and EC accounting for 74. We observed a positive correlation between the use of EC and respiratory symptoms across all categories of cigarette smoking status. Compared to non-users of any type of cigarette, individuals who smoked combustible cigarettes had a 23% higher risk for developing incident respiratory symptoms (RR = 1.23, 95% CI 1.03–1.43). Current EC users were associated with a 28% increased risk for developing incident respiratory symptoms after 5-year follow-up (RR = 1.28, 95% CI 1.01–1.55). In comparison to non-users, those who engaged in dual use of combustible cigarettes and EC had a 41% greater risk for experiencing the outcome of interest (RR = 1.41, 95% CI 1.19–1.61). (Table 2).

**Table 2:** The associations between smoking combustible cigarettes, using electronic cigarettes, and the risk of incident respiratory symptoms in a sample of adults from 2015 to 2020.

	Model 1^♭^	Model 2^¶^	Model 3^♮^
Non-users	1.00	1.00	1.00
Combustible cigarette users	**1.17(1.03–1.32)**	**1.19(1.01–1.35)**	**1.23(1.03–1.43)**
EC users	1.26(0.99–1.53)	1.23(0.98–1.48)	**1.28(1.01–1.55)**
Dual users	**1.46(1.27–1.68)**	**1.43(1.20–1.67)**	**1.41(1.19–1.61)**
*p*-value	< 0.001	< 0.001	< 0.001

Non-users: individuals who never consumed any cigarette products; Dual users: individuals who concurrently engage in the consumption of both electronic cigarettes and combustible cigarettes.

Significant values are in [bold].

EC, electric cigarette.

^♭^Model 1: Unadjusted OR with 95% CI.

^¶^Model 2: Adjusted for the age, gender, education level, drinking status and married status.

^♮^Model 3: Adjusted for the age, gender, education level, drinking status and married status as well as values of physical measurement.

**Figure 2:**
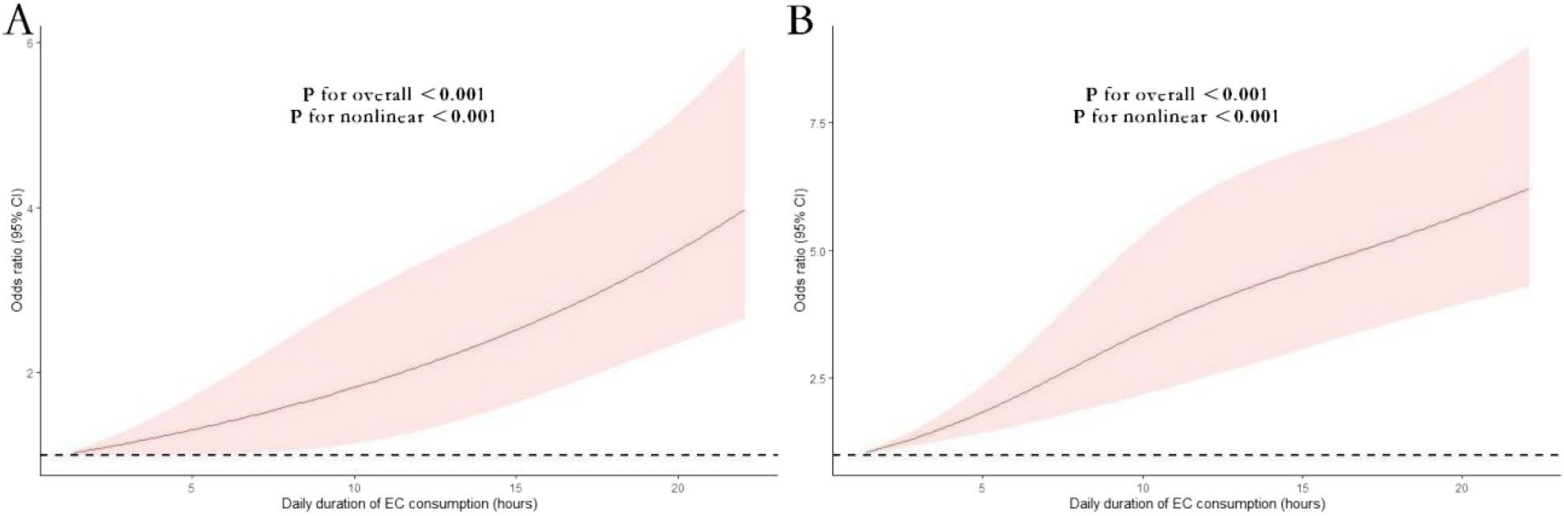
Nonlinear association between daily EC usage by hours and the risk of respiratory symptoms among EC users (**A**) and dual users of combustible cigarettes and EC (**B**). Associations were assessed using logistic regression models with restricted cubic splines.

A non-linear relationship is observed in Fig. 2, illustrating the association between daily EC usage by hours and the risk of respiratory symptoms. Notably, a statistically significant J-shaped pattern is found in this association.

### Association of EC and COPD

Among the 146 incident cases of COPD, non-users accounted for 41 (28.1%), combustible cigarette users constituted 85 (58.2%), EC users comprised 5 (3.4%) cases, and dual users of combustible cigarettes and EC represented 15 (10.3%) cases. Comparatively, combustible cigarette use was associated with a higher risk of incident COPD compared to non-users (OR = 1.07, 95% CI 1.00–1.25). Current EC users exhibited an increased risk of developing incident respiratory symptoms after 5-year follow-up by approximately 8% (OR = 1.08, 95% CI 1.02–1.64). In contrast to non-users, individuals who engaged in dual use of combustible cigarettes and EC had an elevated risk for experiencing the outcome of interest by approximately 18% (RR = 1.18, 95% CI 1.01–1.39). (Table 3).

**Table 3:** The associations between smoking combustible cigarettes, using electronic cigarettes, and the risk of incident COPD in a sample of adults from 2015 to 2020.

	Model 1^♭^	Model 2^¶^	Model 3^♮^
Non-users	1.00	1.00	1.00
Combustible cigarette users	1.08(0.96–1.24)	**1.06(1.01–1.31)**	**1.07(1.00–1.25)**
EC users	**1.10(1.00–1.69)**	1.11(0.98–1.73)	**1.08(1.02–1.64)**
Dual users	1.16(0.99–1.43)	**1.15(1.00–1.36)**	**1.18(1.01–1.39)**
*p-*value	< 0.001	< 0.001	< 0.001

Non-users: individuals who never consumed any cigarette products; Dual users: individuals who concurrently engage in the consumption of both electronic cigarettes and combustible cigarettes.

Significant values are in [bold].

EC, electric cigarette.

♭Model 1: Unadjusted OR with 95% CI.

^¶^Model 2: Adjusted for the age, education level, drinking status and married status.

♮Model 3: Adjusted for the age, education level, drinking status and married status as well as values of physical measurement.

**Figure 3:**
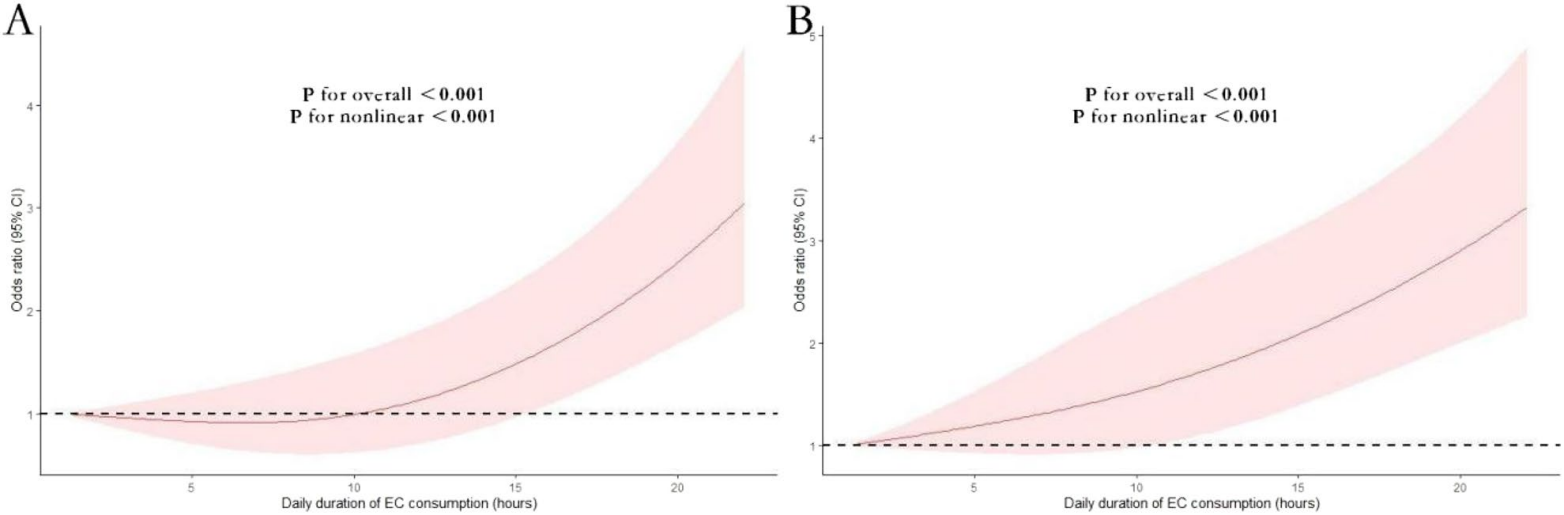
Nonlinear association between daily EC usage by hours and the risk of COPD among EC users (**A**) and dual users of combustible cigarettes and EC (**B**). Associations were assessed using logistic regression models with restricted cubic splines.

The analysis presented in Fig. 3 reveals a non-linear relationship between daily EC usage by hours and the risk of COPD. There is a statistically significant J-shaped pattern is discernible in this association.

### Subgroup and sensitive analyses

**Figure 4:**
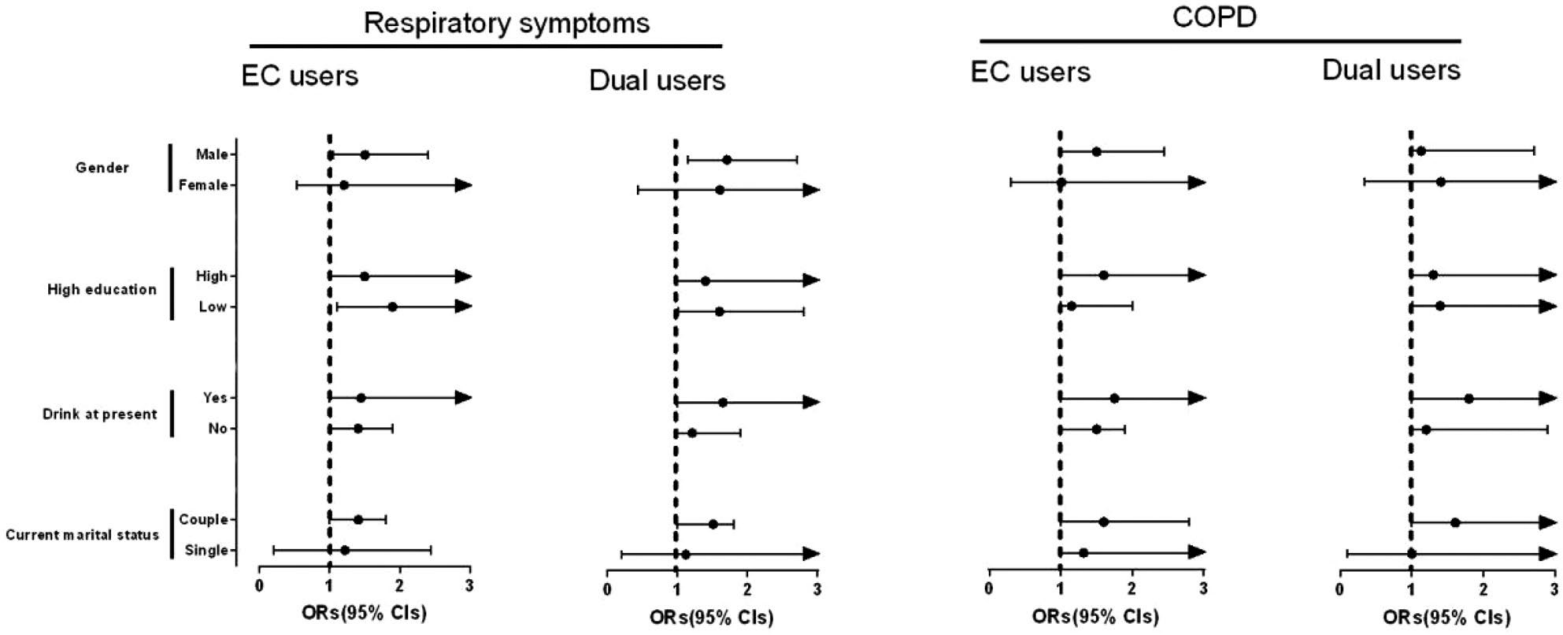
Subgroup analyses of the use of EC and the incidence risk of respiratory symptoms and COPD stratified by all potential risk factors.

The relationship between the use of EC and the incidence risk of respiratory symptoms and COPD, stratified by all potential risk factors, is illustrated in Fig. 4. The subgroup analyses did not reveal any significant alterations in the association between the utilization of EC and the incidence risk of respiratory symptoms and COPD.

The results of sensitivity analyses, as presented in Table 4, demonstrated consistent notable outcomes when re-evaluating the findings after excluding parts of participants.

**Table 4:** Sensitive analyses on the association between smoking combustible cigarettes, using electronic cigarettes, and the incidence of respiratory symptoms as well as COPD from 2015 to 2020.

Health Outcome		Adjusted OR (95%CI)^a^	Adjusted OR (95%CI)^b^	Adjusted OR (95%CI)^c^	Adjusted OR (95%CI)^d^
Respiratory symptoms	Non-users	1.00	1.00	1.00	1.00
	Combustible cigarette users	1.20(0.99–1.50)	**1.22(1.01–1.48)**	**1.28(1.02–1.55)**	1.25(0.98–1.50)
	EC users	**1.29(1.00–1.59)**	**1.23(1.00–1.60)**	**1.31(1.00–1.64)**	**1.30(1.00–1.66)**
	Dual users	**1.35(1.11–1.64)**	**1.40(1.09–1.62)**	**1.39(1.13–1.66)**	**1.40(1.11–1.66)**
	*p-*value	< 0.001	< 0.001	< 0.001	< 0.001
COPD	Non-users	1.00	1.00	1.00	1.00
	Combustible cigarette users	1.05(0.95–1.29)	**1.06(1.00–1.31)**	1.03(0.91–1.30)	1.05(0.90–1.33)
	EC users	1.05(0.98–1.68)	**1.08(1.00–1.71)**	**1.10(1.00–1.67)**	**1.08(1.00–1.65)**
	Dual users	**1.15(1.01–1.42)**	**1.16(1.01–1.43)**	**1.16(1.03–1.49)**	**1.16(1.01–1.48)**
	*p-*value	0.017	< 0.001	< 0.001	< 0.001

Non-users: individuals who never consumed any cigarette products; Dual users: individuals who concurrently engage in the consumption of both electronic cigarettes and combustible cigarettes.

Significant values are in [bold].

COPD, chronic obstructive pulmonary disease; CI, confidence interval.

^a^We excluded the participants who reported “unable to answer” in the questionnaires from the participants. Adjusted for the age, gender,education level, drinking status and married status as well as values of physical measurement.

^b^We excluded the participants who reported their age more than 55 years at baseline. Adjusted for the age, gender, education level, drinking status and married status as well as values of physical measurement.

^c^We excluded the participants who reported other diseases history except for respiratory diseases. Adjusted for the age, gender, education level, drinking status and married status as well as values of physical measurement.

^d^We excluded the participants who reported a history of combustible cigarette smoking. Adjusted for the age, gender, education level, drinking status and married status as well as values of physical measurement.

## Discussion

Our findings indicate a significant correlation between the consumption of ECs and an increased risk of reporting respiratory symptoms and COPD. Moreover, individuals who use both ECs and combustible cigarettes have a higher risk of developing respiratory symptoms and COPD. The relationship between daily EC consumption and the development of respiratory symptoms, as well as COPD, follows a significant J-shaped pattern. Therefore, careful consideration is necessary due to the potential adverse association between the use of ECs, especially when combined with combustible cigarettes, and the development of respiratory symptoms and COPD.

The consumption of EC has witnessed a significant surge in China over the past few years. The prevalence of EC usage among Chinese adults, especially within the age group of 15–24, has observed a notable surge since 2015, as highlighted by the China Project of Global Adults Tobacco Survey^33^. Initially, ECs were proposed as an alternative method for smoking cessation instead of combustible cigarettes, and they did indeed play a certain role in facilitating quitting smoking^34^. However, it was observed that there is a high prevalence of dual use of ECs and combustible cigarettes among individuals attempting to quit smoking, leading to lower success rates in achieving abstinence. Furthermore, the use of ECs has emerged as a potential gateway to combustible cigarette use among adolescents and young adults^31,33^. Consequently, smoking cessation professionals have gradually come to realize that ECs may not possess the same efficacy for promoting cessation as combustible cigarettes. An increasing number of researchers have discovered that the use of ECs is associated with the development of lung injury^35,36^, leading to respiratory diseases^12–15,35^, particularly respiratory symptoms and COPD. Li et al. found a significantly higher association between vaping 2–10 times in adults and ever wheezing compared to those who never vaped (adjusted OR = 1.4, 95%CI 1.1–1.6)^37^. After controlling for covariates, another study reported a significant association between EC use and chronic pulmonary disorder (adjusted OR = 2.58, 95%CI 1.36–4.89, *p* < 0.01)^38^. Notably, there has been a rapid increase in adolescent EC use worldwide despite low rates of regular use in China. Adolescents are able to access and utilize ECs easily and may become regular users within a short period^39^. However, respiratory symptoms are not limited to adult EC users; adolescents who use ECs also exhibit an increased risk. The former study demonstrated that adolescent EC users had a twofold risk of developing chronic respiratory symptoms compared to non-users, and this risk remained almost twofold among past EC users^40^. A recent study conducted on American adolescents additionally suggested that using ECs was associated with bronchitic symptoms (OR = 1.56, 95% CI 1.37–1.77) and shortness of breath (OR = 1.68, 95% CI 1.35–2.08) compared with non-users^41^. Another more severe respiratory disease caused by ECs is COPD. A recent comprehensive review demonstrated a significant association between EC use and the incidence of COPD, even after adjusting for cigarette smoking status and relevant covariates (pooled OR = 1.49, 95% CI 1.36–1.65)^42^. Subsequent research has further supported this positive association between EC use and incident COPD. Bircan et al. reported that EC users had higher odds of self-reported COPD compared to individuals who never used ECs (OR = 1.44, 95% CI 1.42–1.46)^43^. Results from a longitudinal cohort analysis revealed that using ECs independently increased the risk of developing COPD, in addition to combustible tobacco smoking; furthermore, dual use of both ECs and combustible cigarettes was associated with a 3.30-fold higher risk of incident COPD compared to individuals who never smoked or used ECs^44^. The utilization of linear and nonlinear analyses offered a comprehensive perspective on the trajectory of the relationship between exposure factors and health outcomes. Our study is the first to report on the dose–response relationship between EC usage and the incidence risk of respiratory symptoms and COPD. Importantly, our findings suggest a non-linear association, indicating that an increased risk of respiratory symptoms is closely linked to daily EC consumption, even if it is only for one hour. Moreover, dual users exhibited a heightened risk of developing respiratory symptoms. Similar trends were observed in the incidence of COPD; however, a longer duration of electronic cigarette usage is required to develop this condition. The findings from our study, in conjunction with these results, indicate a strong correlation between the use of ECs and the development of pulmonary injury, thereby giving rise to respiratory symptoms and COPD. The focus of follow-up efforts to reduce smoking should be directed towards combustible tobacco smokers, while simultaneously exerting control over the increasing number of EC users and individuals who engage in dual use, particularly among young people.

The laboratory experiments consistently demonstrate the potential biological impact of EC usage on the development of respiratory symptoms and COPD. The available laboratory findings indicate that EC use primarily affects respiratory health through four distinct biological mechanisms: cellular toxicity, heightened oxidative stress, increased vulnerability to infections, and genetic alterations^42^. The concentrations of various pulmonary toxic substances in EC aerosol, including propylene glycol, diacetyl, cinnamaldehyde, benzaldehyde, and metals, are comparatively elevated when compared to their levels in traditional cigarettes^45–47^. The cytotoxic effects and oxidative stress observed were attributed to the repeated exposure of cells to harmful substances released during the heating process of electronic liquid in EC devices^48,49^. The results obtained from experiments conducted on living organisms as well as in laboratory settings indicate that the presence of EC leads to an enhancement in the potency of bacteria and their vulnerability to infections. Additionally, studies using animal models have revealed that exposure to EC aerosol worsens the rates of illness and death associated with both bacterial and viral infections^50,51^. The aerosol from ECs, unlike combusted tobacco smoke, has been found to increase the risk of cell death and DNA damage while also suppressing genes involved in immune function^52^. According to the biological evidence, our study findings demonstrate that the use of ECs independently contributes to an increased risk of respiratory symptoms and COPD. Notably, individuals who engage in dual use of ECs and combustible cigarettes face a higher risk for developing incident respiratory symptoms and COPD compared to those who exclusively use either cigarette type. These findings align with the results observed in our study consensus reports on this topic should be noted as well. It is worth mentioning that dual users of both ECs and combustibles, which represents the most common usage pattern, face an even greater risk for respiratory symptoms and COPD than those who solely use either product alone. Therefore, it is crucial for policymakers responsible for smoking cessation efforts worldwide to pay closer attention to regulating not only individual users of ECs but especially those who engage in dual usage alongside traditional combustible cigarettes according to recommendations made by the World Health Organization^53^. Particularly in countries such as the United States and the United Kingdom, where ECs are more commonly perceived as smoking cessation tools, these nations have placed significant emphasis on the potential of ECs to aid smokers in quitting. In the United States, regulatory oversight of ECs has been undertaken by the Food and Drug Administration (FDA) since 2016, with many states subsequently implementing their own regulations^54^. Similarly, in the United Kingdom, the Medicines and Healthcare products Regulatory Agency (MHRA) has been regulating ECs as a medicinal product since 2016, allowing them to be prescribed on the National Health Service (NHS) for smoking cessation^55^. In fact, several countries with rigorous tobacco control regulations, such as Australia and Canada, offer policies that are worth considering and referencing. These countries classify ECs as traditional cigarettes and regulate them accordingly as tobacco products. In Australia, ECs are subject to the same laws as traditional cigarettes, prohibiting online sales or advertising and restricting use in public places^56^. Similarly in Canada, strict regulations apply to ECs including a ban on flavored products and a requirement for plain packaging^57^.

## Strengths and limitations

Our study firstly provided evidence of a non-linear association between the use of ECs and the risk of developing respiratory symptoms and COPD in a long-term, prospective cohort. The cohort study design, as widely acknowledged, offers a more robust basis for drawing causal conclusions compared to the cross-sectional study design. In our investigation, we specifically recruited individuals who were initially free of respiratory symptoms and COPD, enabling us to realistically explore the association between EC use and the risk of health events. To a certain extent, this study provided long-term evidence of the association between EC use and the incidence of respiratory symptoms and COPD. Furthermore, we examined this association using a large dataset with sufficient statistical power to allow for stratification based on combustible cigarette use status.

Nevertheless, our study does have certain limitations. Firstly, the participant information was collected from a specific occupational population and limited to one province in China. It is worth noting that the Han ethnicity constitutes over 90% of the Chinese population, and thus more than 98% of our participants were Han Chinese. Consequently, they share similar cultural backgrounds and lifestyles, making them a representative sample of the Chinese population. A further limitation was the presence of self-reported bias. As mentioned in the methodology section, participants themselves provided information on their smoking habits, use of EC, and respiratory symptoms. The reliance on retrospective questionnaires for self-reported data may inevitably lead to an increase in misestimated rates. Lastly, despite our efforts to adjust for relevant covariates, it is important to note that not all potential confounders could be accounted for in this study.

## Conclusions

The data derived from an extensive and representative user group of ECs suggests the potential occurrence of EC-associated lung injury in relation to respiratory symptoms and COPD. Individuals who engage in both ECs and combustible cigarettes simultaneously are at a significantly elevated risk of developing new-onset respiratory symptoms and COPD. Policymakers should exercise caution when considering the utilization of ECs as a smoking cessation tool for combustible cigarettes.

## Data Availability

The datasets used during the current study available from the corresponding author on reasonable request.
